# The Multifaceted Role of VIRMA, a Core Component of the Methyltransferase Complex, in Cancer and Cancer Therapy

**DOI:** 10.3390/biom15070912

**Published:** 2025-06-22

**Authors:** Jinmeng Lu, Chengyu Zhang, Mengshuang Yin, Huili You, Chao Xiong, Jing Wu, Ying Gong, Zhangang Xiao, Jing Shen

**Affiliations:** 1 Laboratory of Molecular Pharmacology, Department of Pharmacology, School of Pharmacy, Southwest Medical University, Luzhou 646000, China; xlu15213078380@163.com (J.L.); zcy2129848059@163.com (C.Z.); yms18384007760@163.com (M.Y.); yoongiyhl@163.com (H.Y.); 15183087230@163.com (C.X.); wjhzhf@163.com (J.W.); gloria_gy0417@163.com (Y.G.); 2 Cell Therapy & Cell Drugs of Luzhou Key Laboratory, Luzhou 646000, China; 3 South Sichuan Institute of Translational Medicine, Luzhou 646000, China

**Keywords:** VIRMA, m6A, cancer, m6A independent

## Abstract

VIRMA (also known as KIAA1429), as a core regulatory subunit of the m6A methyltransferase complex, plays a key role in tumorigenesis and progression by dynamically regulating RNA methylation modifications. Studies have shown that VIRMA is aberrantly overexpressed in more than 20 types of malignant tumors, including liver cancer, breast cancer, and lung cancer, and is significantly associated with chromosome 8q amplification and poor prognosis. Its mechanism of action involves regulating the expression of tumor-associated genes through both m6A-dependent and m6A-independent pathways, thereby promoting tumor proliferation, metastasis, and drug resistance. These findings suggest that VIRMA has the potential to serve as a pan-cancer diagnostic and prognostic biomarker. This review summarizes the role of VIRMA in malignant tumors from multiple perspectives and explores its potential applications in clinical diagnosis and treatment.

## 1. Introduction

According to the GLOBOCAN 2024 database, there were 20 million new cancer cases and 9.7 million cancer-related deaths globally in 2022. It is projected that by 2050, the number of new cancer cases will exceed 35 million, and cancer may become the leading cause of death in every country within the 21st century [1]. The occurrence and progression of tumors are often closely associated with changes in gene expression. In addition to DNA sequence alterations, gene expression is regulated at multiple levels, including epigenetic (such as DNA and histone modifications), transcriptional, epitranscriptome (RNA modification), and translational regulation [2]. In recent years, research on RNA modifications has rapidly advanced. Currently, more than 170 types of RNA post-transcriptional modifications are known, with N6-methyladenosine (m6A) being the most abundant endogenous RNA modification in eukaryotes [3,4].

m6A RNA methylation is a methylation reaction that occurs at the N6 position of adenosine. In mRNA, m6A sites are highly enriched in the 5′ untranslated region (5′-UTR), near the stop codon, in the 3′ untranslated region (3′-UTR), and in long introns [5,6]. This modification is dynamically reversible and is mediated by three classes of enzymes: “writers” (methyltransferases), “erasers” (demethylases), and “readers” (m6A-binding proteins). m6A deposition is primarily carried out by the “writer” methyltransferase complex [7,8]. “Erasers” are demethylases that make the m6A modification process reversible [9]. “Readers” are m6A-binding proteins that specifically recognize m6A-modified RNAs and regulate their function [10]. These proteins regulate RNA function and expression, significantly affecting cancer initiation and progression [11,12].

The methyltransferase complex consists of methyltransferase-like 3 (METTL3), methyltransferase-like 14 (METTL14), and auxiliary factors such as Wilms’ tumor 1-associating protein (WTAP), RNA-binding motif protein 15 (RBM15), ZC3H13, RBM, HAKAI, VIRMA, and others, with a molecular weight of approximately 1000 kDa [13]. Most of these components have been shown to participate in tumorigenesis and progression, such as METTL3 and WTAP [14,15,16]. As the largest structural component of the m6A methyltransferase complex, VIRMA (KIAA1429) serves as a scaffold protein that recruits the catalytic core (METTL3-METTL14) to RNA substrates and maintains methylation activity under specific conditions [13,17]. Given its central role in m6A modification, VIRMA has garnered significant attention in recent years. Accumulating evidence demonstrates that VIRMA is markedly overexpressed in multiple malignancies (e.g., hepatocellular carcinoma, breast cancer, colorectal cancer), where it drives tumor proliferation, invasion, and metastasis through m6A-dependent mechanisms [18,19,20]. Notably, emerging studies reveal m6A-independent functions of VIRMA, expanding our understanding of its oncogenic regulatory networks [21]. These findings position VIRMA not only as a promising diagnostic biomarker but also as a novel therapeutic target. This review systematically summarizes current advances in VIRMA research, focusing on its molecular functions, regulatory mechanisms, and clinical translation potential, thereby providing new perspectives for m6A-related research and precision cancer therapy.

## 2. Localization, Structure, and Function of VIRMA

*VIRMA* (Vir-like m6A methyltransferase associated), also known as *KIAA1429*, is a gene located at 8q22.1 in humans and contains 25 exons. This gene encodes two distinct isoforms: a full-length isoform containing 1812 amino acids (202 kDa) and an N-terminal truncated isoform consisting of the first 1130 amino acids. Current evidence indicates that the full-length isoform predominantly mediates oncogenic functions in malignancies, while the biological role and regulatory mechanisms in polyadenylation site selection of the short isoform remain largely unexplored [22]. The subcellular distribution of VIRMA protein is closely related to tumor type and occurrence. In most malignancies (e.g., pancreatic ductal adenocarcinoma [PDAC] and hepatocellular carcinoma [HCC]), VIRMA primarily localizes to the nucleus. However, studies in breast cancer (BC) demonstrate predominant cytoplasmic localization, where it regulates the stability of downstream target HAS2. This dynamic nuclear-cytoplasmic distribution strongly correlates with tumor malignancy and metastatic potential, suggesting VIRMA localization may serve as a potential tumor grading biomarker [19,23,24]. However, the precise mechanisms governing VIRMA’s subcellular trafficking and its context-dependent functional variations in different tumor microenvironments require further investigation.

**Figure 1:**
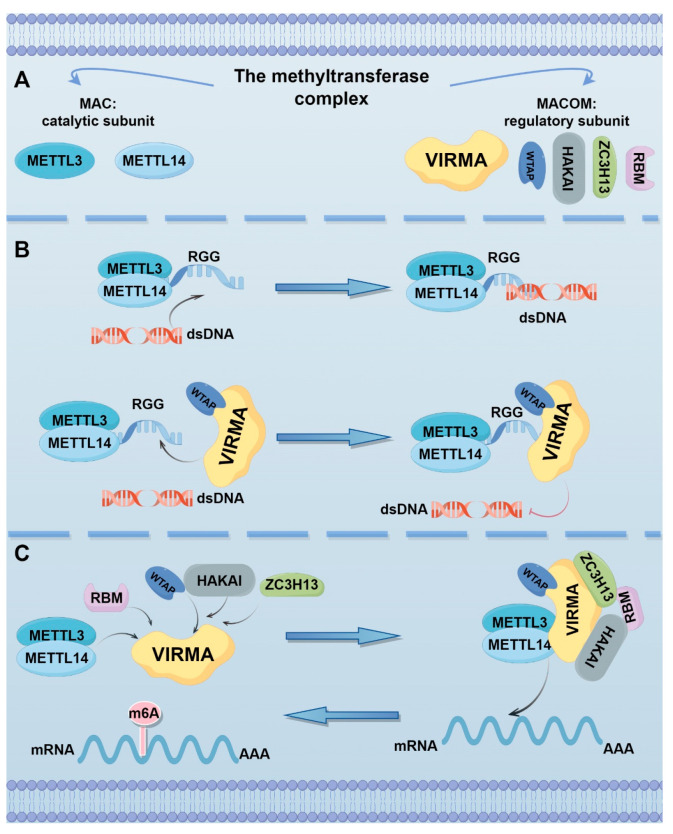
The role of VIRMA in m6A methylation. (**A**) Components of the methyltransferase complex. (**B**) dsDNA inhibits the RNA methyltransferase activity of METTL3-METTL14. WTAP-VIRMA binds to the RGG motif, preventing dsDNA from interfering and thereby maintaining the RNA methylation activity of METTL3-METTL14. (**C**) VIRMA acts as a scaffold for the methyltransferase complex, recruiting the core catalytic components and linking them to the RNA substrate.

Current research on VIRMA primarily focuses on its role as a key component of the m6A methyltransferase complex. This complex structurally consists of two functional units: (1) the catalytic core MAC (m6A-METTL complex), formed by the METTL3-METTL14 heterodimer that directly catalyzes m6A modification; and (2) the regulatory complex MACOM (m6A-METTL associated complex), composed of WTAP, VIRMA, ZC3H13, and HAKAI, which mediates scaffold assembly, substrate recruitment, complex stabilization, and subcellular localization. As a core structural component of MACOM, VIRMA plays an essential role in bridging the catalytic core with regulatory subunits (Figure 1A) [25]. In cryo-EM structural analysis, VIRMA (residues 334–1585), WTAP (residues 64–247), and ZC3H13 (residues 1492–1643) together form the “war horse”-shaped human MACOM core. VIRMA adopts a horseshoe-like conformation and contains 20 ARML modules, with its surface charges predominantly negative and a few small areas carrying a positive charge. These regions are relatively conserved, confirming that VIRMA may play an important role in the interaction with other components of MACOM, particularly WTAP [26].

The RGG motif is a common protein sequence of arginine-glycine residues that mediates nucleic acid binding or protein-protein interactions [27]. Yan et al. found that the RGG motif can directly bind with WTAP-VIRMA and facilitate the binding of METTL3-METTL14 to RNA and double-stranded DNA (dsDNA). However, the presence of dsDNA significantly inhibits the RNA methyltransferase activity of METTL3-METTL14. As a regulatory subunit, WTAP-VIRMA maintains the RNA methylation activity of METTL3-METTL14 by preventing dsDNA binding through their interaction with the RGG motif. Consequently, the methylation activity of the METTL3-METTL14-WTAP-VIRMA quaternary complex (M3-M14-W-V) is significantly higher than that of the METTL3-METTL14 dimer (Figure 1B). Therefore, downregulation of either WTAP or VIRMA significantly affects the overall level of m6A in mRNA [17]. Additionally, there is evidence that VIRMA can serve as a scaffold for the methyltransferase complex, recruiting catalytic core components and linking them with RNA substrates (Figure 1C). After knockdown of VIRMA in A549 cells, the m6A peak score decreased by four times, far exceeding the reduction observed with METTL3 or METTL4 knockdown alone [13,28].

RNA-binding proteins (RBPs) are proteins with specific RNA-binding domains that can form various dynamic ribonucleoprotein complexes with RNA molecules, regulating multiple aspects of gene expression, including RNA splicing, mRNA stability, mRNA localization, and translation [29]. In Drosophila, the virilizer, which is homologous to KIAA1429, contains RBP structural domains similar to those found in RNA and DNA helicases, translation initiation factor 2 (IF2), and ribonucleoproteins [30]. Therefore, VIRMA may exert its biological functions independently of methylation modification.

## 3. The Role of VIRMA in Cancer

**Figure 2:**
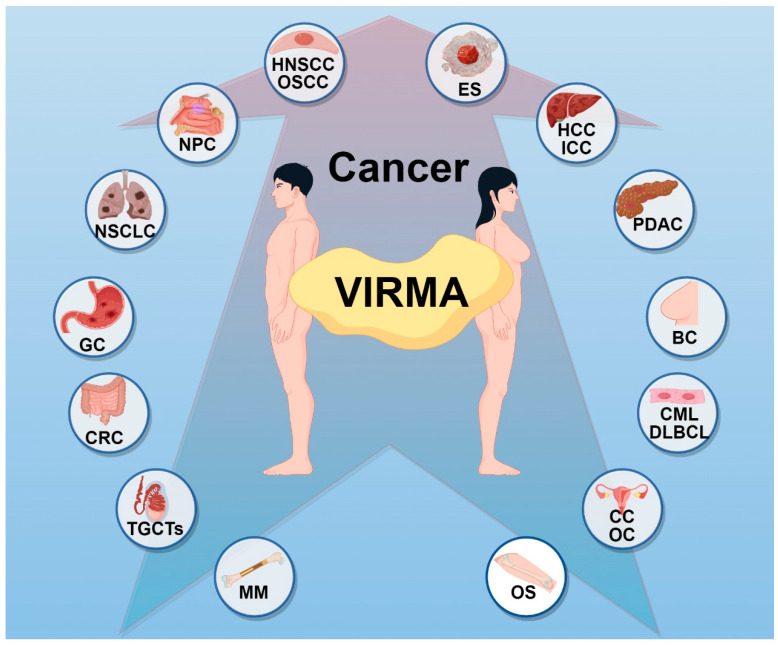
VIRMA is upregulated in eighteen types of malignant tumors. Elevated VIRMA expression has been documented across a wide spectrum of malignancies. HNSCC, head and neck squamous cell carcinoma; NPC, nasopharyngeal carcinoma; OSCC, oral squamous cell carcinoma; NSCLC, non-small cell lung cancer; HCC, hepatocellular carcinoma; ICC, intrahepatic cholangiocarcinoma; GC, gastric cancer; PDAC, pancreatic ductal adenocarcinoma; CRC, colorectal cancer; MM, multiple myeloma; OS, osteosarcoma; DLBCL, diffuse large B-cell lymphoma; CML, chronic myeloid leukemia; BC, breast cancer; TGCTs, testicular germ cell tumors; CC, cervical cancer; OC, ovarian cancer; and ES, Ewing’s sarcoma.

Among all 15 core m6A genes, *VIRMA* exhibits the highest frequency of abnormalities, with 21.4% of tumor samples showing expression or copy number alterations (e.g., overexpression or amplification), significantly higher than METTL3 (6.2%) [31]. Numerous studies have shown that dysregulation of VIRMA is closely related to the initiation and progression of malignancies. Elevated VIRMA expression has been observed in eighteen types of malignancies, including head and neck squamous cell carcinoma (HNSCC), nasopharyngeal carcinoma (NPC), oral squamous cell carcinoma (OSCC), non-small cell lung cancer (NSCLC), hepatocellular carcinoma (HCC), intrahepatic cholangiocarcinoma (ICC), gastric cancer (GC), PDAC, colorectal cancer (CRC), multiple myeloma (MM), osteosarcoma (OS), diffuse large B-cell lymphoma (DLBCL), chronic myeloid leukemia (CML), BC, testicular germ cell tumors (TGCTs), cervical cancer (CC), ovarian cancer (OC), and Ewing’s sarcoma (ES) (Figure 2) [18,19,24,32,33,34,35,36,37,38,39,40,41,42,43,44,45]. In recent years, research on VIRMA has been increasing, making it particularly important to comprehensively summarize its role in cancer. The following sections will provide a detailed overview of the functions and mechanisms of VIRMA as an m6A writer, including promoting cancer cell proliferation and metastasis, influencing cell cycle, affecting cancer metabolism, mediating resistance to ferroptosis, mediating immune escape, and affecting drug resistance, as well as its involvement in tumor progression through m6A-independent mechanisms (Table 1).

### 3.1. Promoting Cancer Cell Proliferation and Metastasis

**Figure 3:**
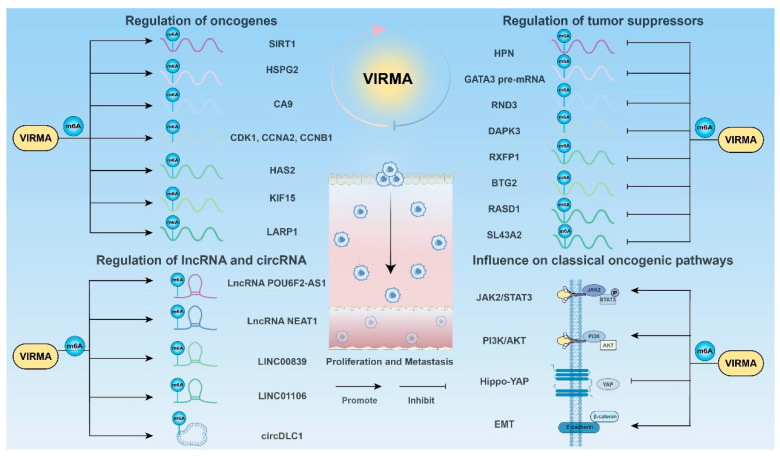
VIRMA promotes cancer cell proliferation and metastasis. VIRMA upregulates oncogenes, downregulates tumor suppressors, and modulating lncRNAs, circRNAs, and classical oncogenic pathways to promote cancer cell proliferation and metastasis in an m6A-dependent manner.

Tumor metastasis refers to the spread of tumor cells from the primary site to distant organs or tissues, leading to the formation of new tumor lesions. Invasion and metastasis are among the most lethal characteristics of cancer, with over 90% of cancer-related deaths being closely linked to tumor metastasis. Therefore, a deeper understanding of the molecular mechanisms under metastasis is of significant importance for preventing cancer-related death (Figure 3) [87,88,89].

#### 3.1.1. Regulation of Oncogenes

CCL3 (Macrophage Inflammatory Protein-1 alpha, MIP-1α) belongs to the C-C chemokine family and is involved in the occurrence and development of various malignant tumors [90]. Zhou et al. discovered through co-culturing normal liver cells with ICC cells that CCL3 secreted by normal liver cells was significantly increased, further upregulating VIRMA expression, leading to an overall increase in m6A levels and enhancing the invasion and metastasis abilities of tumor cells. MeRIP-seq analysis showed that VIRMA enhanced the expression of SIRT1 through an m6A methylation-dependent mechanism, promoting the progression of ICC [69]. Chronic hepatitis B virus (HBV) infection plays a critical role in the initiation and progression of HCC. The hepatitis B virus X protein (HBx) is recognized as the sole regulatory protein encoded by the HBV genome, which promotes viral replication by stimulating *HBV* gene expression. In HCC cells with stable HBx expression, HBx upregulated VIRMA expression, thereby driving m6A methylation of its target gene *HSPG2*. This enhanced the stability of HSPG2 and subsequently promoted the proliferation, migration, and survival of HCC cells [66].

Inhibin A (INHBA) belongs to the transforming growth factor-β superfamily and is closely associated with cancer cell invasion, proliferation, and metastasis [91]. In OSCC, INHBA participates in tumorigenesis through VIRMA-regulated m6A modification [48]. CA9, a transmembrane zinc metalloprotease with cell adhesion functions, is crucial for tumor growth and survival. Overexpression of CA9 in various cancers is associated with poor prognosis. VIRMA upregulated CA9 expression in an m6A-dependent manner in OSCC, and inhibition of VIRMA suppressed tumor growth both in vitro and in vivo [49].

In OS, silencing VIRMA resulted in a reduction of overall m6A methylation level in SW1353 cells, inhibiting their proliferative capacity. Bioinformatic analysis indicated that VIRMA may promote OS progression by targeting CDK1, CCNA2, and CCNB1 [76].

In NPC, VIRMA mediated m6A modification of PTGS2, enhancing its mRNA stability and promoting cancer progression [46].

In HNSCC, VIRMA facilitated cancer progression by modulating the m6A levels of UBR5 [35]. The expression of the oncogene *SIRT1* was positively correlated with VIRMA in CRC. VIRMA upregulated SIRT1 mRNA stability through an m6A-dependent mechanism, thereby promoting the growth of CRC cells [20].

Hyaluronan synthase 2 (HAS2) plays a critical role in both normal development and tumorigenesis. Studies have shown that VIRMA, by acting as a scaffold protein, recruited the m6A reader IGF2BP3 to increase the m6A modification level of HAS2, stabilizing its mRNA and mediating BC cell proliferation and migration [19]. Additionally, VIRMA enhanced the mRNA stability of the pro-oncogenic gene *KIF15* through an m6A-dependent mechanism, thereby promoting BC progression [78].

In CC, VIRMA increased the mRNA stability of LARP1, contributing to the initiation and progression of CC [82].

#### 3.1.2. Regulation of Tumor Suppressors

Hepsin (HPN) is a transmembrane serine protease, and its expression is decreased in HCC. A study by Meng et al. demonstrated that overexpressing HPN could effectively inhibit the invasion, migration, and proliferation of HCC cells. VIRMA inhibited the expression of HPN in an m6A-dependent manner, further promoting HCC progression [60]. Additionally, VIRMA could increase the m6A modification level of ID2 mRNA, suppress its expression, and facilitate migration and invasion in HCC [62]. GATA3 is a well-established tumor-suppressive transcription factor. Also in HCC, VIRMA also mediated the m6A modification of GATA3 pre-mRNA, inhibited its binding to the RNA-binding protein HuR, thus reduced the stability of GATA3 pre-mRNA, and ultimately led to downregulation of GATA3 expression. Moreover, the antisense long non-coding RNA GATA3-AS interacted with VIRMA and GATA3 pre-mRNA, guiding VIRMA to preferentially mediate the m6A modification of GATA3 pre-mRNA [23,92]. Another study demonstrated that VIRMA downregulated the mRNA stability of the tumor suppressor RND3 through an m6A-YTHDC1-dependent mechanism, thereby suppressing its expression and exerting oncogenic effects in HCC cells [18].

In NSCLC, VIRMA mediated the m6A modification of DAPK3, facilitating YTHDF2/3-mediated DAPK3 post-transcriptional degradation and promoting tumor growth. DAPK3 (Death-Associated Protein Kinase 3) silencing can reverse the invasion and migration suppression caused by VIRMA depletion [53].

RXFP1 (Relaxin Family Peptide Receptor 1) overexpression inhibits the proliferation, migration, and invasion of cancer cells. VIRMA could regulate the m6A modification level of RXFP1, suppressing its expression and exerting oncogenic effects in NSCLC [50]. VIRMA also regulated the mRNA stability of the tumor suppressor gene *BTG2* through an m6A-YTHDF2-dependent mechanism, suppressing its expression and promoting the progression of lung adenocarcinoma (LUAD) [52].

A similar mechanism was observed in CC, where VIRMA negatively regulated the mRNA stability of BTG2 via an m6A-YTHDF2-dependent pathway, thereby facilitating malignant tumor progression [44]. RASD1 has been implicated as a potential tumor suppressor in GC. VIRMA reduced the mRNA stability of RASD1 through an m6A-YTHDF2-dependent mechanism, promoting GC development [36].

In PDAC, alcohol activated the transcription factor C/EBPβ, which bound to the promoter of VIRMA and enhanced its transcription. VIRMA further accelerated the degradation of SLC43A2 mRNA via the m6A-YTHDF2 pathway, reducing phenylalanine uptake and oxidative stress in cancer cells, ultimately leading to cancer progression [73].

#### 3.1.3. Regulation of lncRNA

Long non-coding RNAs (lncRNAs) are defined as non-coding transcripts longer than 200 nucleotides. In recent years, the functions of lncRNAs in tumor initiation and progression have attracted extensive research interests [93]. Similar to mRNA, VIRMA can also mediate m6A methylation of lncRNA.

LncRNA POU6F2-AS1 is upregulated in CRC, and inhibiting this lncRNA suppresses the malignant phenotype of cancer cells. Studies have demonstrated that VIRMA increased the expression of POU6F2-AS1 through m6A modification, promoting the development of CRC [74]. LncRNA NEAT1 has oncogenic potential in various types of cancer. Lee et al. found that the full-length isoform of VIRMA (VIRMA FL) was primarily expressed in BC cells and significantly increased m6A levels. Overexpression of VIRMA FL enhanced the m6A methylation level of lncRNA NEAT1, promoting BC cell growth [80,94]. Zheng et al. demonstrated that LINC00839 directly interacted with transcription factor TAF15 and promoted the transcription of AOC1 by recruiting TAF15, thereby increasing the expression of AOC1 and promoting the growth and metastasis of NPC. VIRMA and IGF2BP1 mediated the m6A modification of LINC00839 and enhanced its expression and stability [47]. The level of LINC01106 is closely associated with clinical malignant features of cancer and can serve as a poor prognostic factor. VIRMA was found to enhance the m6A modification and expression of LINC01106 in LUAD, thereby promoting tumor progression [56,95].

#### 3.1.4. Regulation of Circ RNA

Circular RNA (circRNA) is formed by exon or intron backsplicing, characterized by stability, sequence conservation, and expression specificity [96].

Liu et al. identified circDLC1 as the most significantly differentially expressed circRNA in HCC through RNA-seq and m6A-seq analysis of VIRMA-regulated circRNAs. Overexpression of circDLC1 inhibited the proliferation and metastasis of HCC cells. Moreover, circDLC1 was negatively correlated with VIRMA and positively correlated with patient prognosis. circDLC1 interacted with the RNA-binding protein HuR, preventing HuR from binding to MMP1 mRNA, thereby reducing the expression of MMP1 [61].

#### 3.1.5. Influence on Classical Oncogenic Pathways

VIRMA not only regulates tumor-associated proliferation and metastasis factors but also participates in the activation of classical oncogenic signaling pathways, such as STAT3 and PI3K/AKT, which are crucial for cancer proliferation and metastasis. The JAK2/STAT3 signaling pathway, a member of the STAT family of transcription factors, is closely associated with the onset of various cancers and regulates cancer cell proliferation and migration [97].

ES is a highly invasive bone and soft tissue cancer that primarily affects children and young adults. Downregulation of VIRMA expression significantly inhibits ES proliferation. Bioinformatic analysis showed that NKX2-2 regulates VIRMA-associated m6A writers, promoting a positive feedback loop between VIRMA and STAT3, thus enhancing the malignant phenotype of ES [77,98].

The dysregulation of the PI3K/AKT pathway is closely linked to cancer progression [99]. VIRMA was found to enhance the activity of the PI3K/AKT pathway by upregulating the m6A modification of the tumor suppressor ARHGAP30, promoting lung cancer cell proliferation and migration [55]. In NPC, VIRMA maintained the stable expression of the transcription factor E2F7 through an m6A-dependent mechanism. E2F7 bound with CBFB, activating the PI3K-AKT signaling pathway and promoting tumorigenesis [32].

The Hippo-YAP signaling pathway is another important pathway involved in cancer. VIRMA was found to mediate the m6A modification of CHST11 mRNA and reduce its expression via an m6A-YTHDF2-dependent mechanism. The interaction between CHST11 and the core kinase MOB1B of the Hippo pathway was therefore disrupted, inhibiting the activation of the Hippo-YAP signaling pathway and thereby promoting tumor development [41].

Epithelial-mesenchymal transition (EMT) is a biological process defined by morphological changes and characteristic markers (such as E-cadherin and vimentin) and plays a critical role in the invasion and metastasis of tumors [100]. In ICC, VIRMA enhanced the expression of downstream targets TMED2 and PARD3B via an m6A-Hur-dependent mechanism. The increased levels of TMED2 and PARD3B led to activation of the AKT/GSK/β-catenin and MEK/ERK/Slug signaling pathways, promoting the expression of EMT markers vimentin and N-cadherin while inhibiting E-cadherin expression, thereby promoting the metastasis and EMT of ICC [68].

### 3.2. Influencing Cell Cycle

Cell cycle dysregulation is the foundation of abnormal cell proliferation in cancer. In most cancers, signaling pathways related to cell proliferation interfere with the normal progression of the cell cycle, thereby contributing to tumor initiation and progression [101].

In DLBCL, overexpression of VIRMA upregulated the anti-apoptotic protein Bcl-2 while inhibiting the expression of pro-apoptotic proteins (such as Cleaved-Caspase 3, Cleaved-Caspase 8, Cleaved-Caspase 9, and Cleaved-PARP), thereby suppressing apoptosis. After VIRMA downregulation, the proportion of DLBCL cells in the G2/M phase significantly increased, with higher expression of the cell cycle inhibitor p21 and lower expression of Cyclin B1. These results indicate that reducing VIRMA expression effectively suppresses DLBCL cell cycle progression [41]. In GC cells, knockdown of VIRMA led to cell cycle arrest in the S phase, increased apoptosis in AGS and HGC-27 cells, and promoted the transition to the G2/M phase [36,86]. In BC, reduced expression of VIRMA also caused a significant delay in the S phase in MCF-7 and SUM1315 cells [83]. Furthermore, in LUAD, VIRMA has been demonstrated to regulate the expression of the oncogene *MUC3A* through an m6A-dependent mechanism and promote cancer progression. The study also showed that downregulation of VIRMA can partially reverse the G1-phase arrest in A549 and H1299 cells with high MUC3A expression [54]. In HNSCC, after VIRMA knockout, most cancer cells were arrested in the S phase, indicating a reduction in the number of dividing tumor cells following VIRMA knockout [35]. The changes in the cell cycle are indirectly caused by the alteration of VIRMA, but the specific mechanism of interaction between VIRMA and cell cycle regulators remains unclear.

Future research may focus on exploring the direct interaction mechanisms between VIRMA and cell cycle-related proteins to deepen the understanding of the connection between VIRMA and cancer.

### 3.3. Affecting Cancer Metabolism

Aerobic glycolysis is the primary energy source for tumor cells’ rapid proliferation, invasion, and metastasis in various cancers and is closely associated with tumor malignancy [102,103]. This metabolic shift from oxidative phosphorylation to aerobic glycolysis is a hallmark of tumor cells and one of the most significant metabolic reprogramming phenomena in cancer cells [104].

VIRMA regulates tumor metabolic reprogramming by influencing key factors in tumor cell aerobic glycolysis. Glucose transporter-1 (GLUT1) plays a critical role in aerobic glycolysis and is closely related to tumor progression and tumor size [105]. In GC, VIRMA could interact with the m6A modification site of LINC00958, promoting its enrichment and enhancing its binding with GLUT1 mRNA, thereby increasing the stability of GLUT1 and ultimately promoting aerobic glycolysis in GC cells [70]. Hexokinase (HK) is the first rate-limiting enzyme in the glycolytic pathway, with major isoforms including HK1 and HK2 [106]. In liver cancer, upregulation of VIRMA significantly promoted lactate production, ATP synthesis, and glucose uptake. VIRMA enhanced HK1 mRNA stability through an m6A-dependent mechanism, increasing HK1 protein level and strengthening the first step of glycolysis, which provides the material basis for the Warburg effect. Inhibiting VIRMA reduced the Warburg effect and increased the sensitivity of liver cancer cells to sorafenib [63]. Furthermore, Li et al. found that VIRMA increased HK2 mRNA stability in an m6A-dependent manner, promoting aerobic glycolysis in CRC [107]. In PDAC, VIRMA cooperated with IGF2BP2 to enhance STRA6 mRNA stability via an m6A-dependent mechanism, which activates HIF-1α and upregulates GLUT1 and HK2, promoting aerobic glycolysis, cell proliferation, and metastasis [24]. Alpha-enolase (ENO1) is a key metabolic enzyme in the glycolytic pathway. In OC, VIRMA regulates ENO1 mRNA stability via an m6A-dependent mechanism, promoting glycolysis, proliferation, and metastasis in OC cells [45]. In OSCC, VIRMA regulated PGK1 via the YTHDF1/m6A-dependent pathway, leading to increased glucose uptake, lactate production, and extracellular acidification rate (ECAR), with decreased oxygen consumption rate (OCR), ultimately enhancing the glycolytic rate and capacity of cancer cells [33]. In MM, VIRMA expression is significantly higher than in normal tissue and is associated with poor prognosis in patients. The mRNA level of VIRMA positively correlated with the mRNA levels of HK2, ENO1, and lactate dehydrogenase A (LDHA), indicating that VIRMA plays a regulatory role in MM cell aerobic glycolysis. Moreover, VIRMA stabilized FOXM1, a player involved in aerobic glycolysis, in cooperation with YTHDF1, further emphasizing VIRMA’s importance in MM cell metabolism and biological behavior [39].

### 3.4. Mediating Resistance to Ferroptosis

Ferroptosis is a form of non-apoptotic, iron-dependent cell death triggered by the toxic accumulation of lipid peroxides in cell membranes [108]. Because ferroptosis is mechanistically and morphologically distinct from other forms of cell death, such as apoptosis, it has garnered significant attention for its role in cancer in recent years [109]. In cancer, epigenetic alterations can regulate the expression of ferroptosis regulators or related pathways, thus affecting the sensitivity of cells to ferroptosis [110].

The solute carrier family member SLC7A11, the 11th member of this family, can transport extracellular cysteine into cells for cysteine synthesis and the biosynthesis of glutathione. SLC7A11 counters cellular oxidative stress by maintaining intracellular glutathione (GSH) levels, thereby inhibiting ferroptosis [111]. In HCC, when VIRMA was inhibited, levels of lipid peroxides and oxidative C11-BODIPY were significantly increased, while VIRMA overexpression had the opposite effect. Mechanistically, KIAA1429 regulated SLC7A11 through m6A post-transcriptional modification, thereby exerting its ferroptosis-inhibiting role [64]. Wu et al. found that si-VIRMA significantly reduced the viability of NSCLC cells treated with the ferroptosis inducer erastin and increased the levels of reactive oxygen species (ROS), MDA, and Fe^2+^ in the cells while decreasing GSH levels. At the same time, ferroptosis-related proteins such as PTGS2, GPX4, and FTH1 also showed significant changes. These results indicate that VIRMA acts as a ferroptosis inhibitor in NSCLC, and its downregulation can promote erastin-mediated ferroptosis [57]. Similarly, in OSCC, silencing VIRMA upregulated the concentrations of Fe^2+^ and lipid ROS, promoting ferroptosis in cancer cells [33]. In CRC, VIRMA expression was significantly elevated in tumor cells, especially in radioresistant cell lines (such as HCT116R and SW620R), where its expression was further increased. Upon VIRMA knockdown, ferroptosis in tumor cells significantly increased, characterized by elevated intracellular Fe^2+^ levels, increased expression of ferroptosis-related protein ACSL4, and decreased expression of SLC7A11 and GPX4. In addition, intracellular ROS levels were increased, and GSH content was decreased. Mechanistically, VIRMA upregulated LncRNA EBLN3P expression in an m6A-dependent manner, and EBLN3P competed with miR-153-3p to increase VIRMA expression, thereby reducing ferroptosis in tumor cells and enhancing their radioresistance [75].

The m6A modification mediated by VIRMA in regulating ferroptosis is a promising area of further exploration in treating cancer and overcoming cancer resistance [112].

### 3.5. Mediating Immune Escape

The tumor microenvironment has a significant impact on the survival and function of CD8^+^ T cells. A metabolic environment unfavorable to the survival of CD8^+^ T cells accelerates their exhaustion, thus inhibiting tumor immunity, which is a key challenge in cancer immunotherapy [113].

Through analysis of TCGA BC data, Bin Lian et al. found that DDR1, a receptor tyrosine kinase (RTK) family member targeting collagen, is negatively correlated with the proportion of CD8^+^ T cells and CD4^+^ T cells. Silencing DDR1 in BC cells increased the infiltration of CD8^+^ T cells within the tumor by reducing the content of collagen IV in the extracellular matrix (ECM) and disrupting the alignment of collagen fibers. VIRMA mediated m6A modification of TFAP2A, a transcription factor for DDR1, thereby enhancing TFAP2A expression. The upregulation of TFAP2A promoted DDR1 expression by binding to the promoter region, inducing the alignment of collagen fibers, inhibiting the infiltration of immune cells, and accelerating immune escape in BC [79]. Additionally, the PD-1/PD-L1 checkpoint suppresses T cell receptor-mediated cytotoxicity and inhibits the proliferation of CD8+ T cells, thereby accelerating immune escape of tumor cells. *KLF1*, an important oncogene, is regulated by VIRMA in NSCLC through m6A modification. VIRMA could stabilize KLF1 mRNA, upregulate KLF1 expression, and consequently enhance PD-L1 expression and promote immune evasion by tumor cells [58].

Although there is limited research on VIRMA’s role in the immune microenvironment and immune infiltration, existing studies suggest the potential of targeting VIRMA to enhance the effectiveness of immunotherapy.

### 3.6. Affecting Drug Resistance

**Figure 4:**
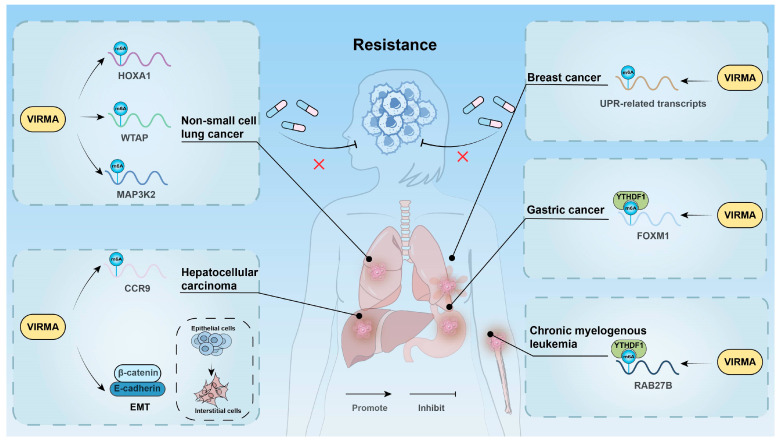
VIRMA regulates cell resistance to chemotherapy through m6A modification.

Chemotherapeutic drugs play a crucial role in treating various cancers by targeting rapidly proliferating tumor cells. However, chemotherapy resistance remains a major cause of poor prognosis in cancer patients [114]. Studies have shown that VIRMA regulates cell resistance to chemotherapy at multiple levels through m6A modification (Figure 4).

In CML, the expression of VIRMA was significantly higher in imatinib-resistant cell lines (K562/G01) compared with sensitive cell lines (K562 and KCL22). VIRMA enhanced the stability of RAB27B mRNA through m6A/YTHDF1, upregulating its expression and promoting CML cell proliferation, thereby inducing imatinib resistance [42]. In LUAD, VIRMA increased the m6A modification level of MAP3K2, activating the JNK/MAPK pathway and inducing gefitinib resistance [34]. Additionally, in non-small cell lung cancer, VIRMA enhanced the m6A modification level of HOXA1, stabilizing its mRNA and promoting tumor cell proliferation and gefitinib resistance [59]. VIRMA could also stabilize WTAP mRNA through an m6A-dependent mechanism, mediating the proliferation and gefitinib resistance of NSCLC cells via the VIRMA/WTAP axis [51]. In liver cancer, the EMT process in sorafenib-resistant cell lines was regulated by m6A methylation. Silencing VIRMA reduced m6A methylation levels, inhibited the invasion, migration, and EMT of sorafenib-resistant cells, and decreased angiogenesis [67]. In hepatitis B virus surface small antigen (SHBs)-induced liver cancer cells, SHBs upregulated VIRMA expression, mediating the m6A modification of CCR9 mRNA and enhancing its stability, thus promoting regorafenib resistance. The m6A modification at positions 1373 and 1496 of CCR9 mRNA is critical for this process [65]. In GC, VIRMA enhanced FOXM1 mRNA stability via an m6A-YTHDF1-dependent mechanism, inducing cisplatin resistance [71]. Additionally, the VIRMA/m6A/FOXM1 axis also played a crucial role in oxaliplatin resistance in GC [72]. In testicular germ cell cancer, knocking out VIRMA reduced the m6A modification level and the expression of components of the methyltransferase complex, significantly inhibiting the survival of cisplatin-resistant cells [43]. Endoplasmic reticulum (ER) stress, triggered by the unfolded protein response (UPR), plays a key role in maintaining tumor survival and chemotherapy resistance, but prolonged UPR activation may also induce tumor cell death [115,116]. In BC, VIRMA upregulation increased the m6A modification level of UPR-related transcripts, enhancing sensitivity to UPR and ER stress, thereby inducing tumor cell death. However, whether the association of VIRMA with UPR/ER stress can enhance chemotherapy sensitivity requires further investigation [80].

Targeting VIRMA in combination with chemotherapeutic drugs may significantly improve drug sensitivity and reverse tumor resistance. Further research into the mechanisms by which VIRMA regulates resistance-related genes through m6A modification will help uncover the underlying mechanisms of tumor resistance and provide new therapeutic strategies for chemotherapy-resistant patients.

### 3.7. Involvement in Tumor Progression Through m6A-Independent Mechanisms

**Figure 5:**
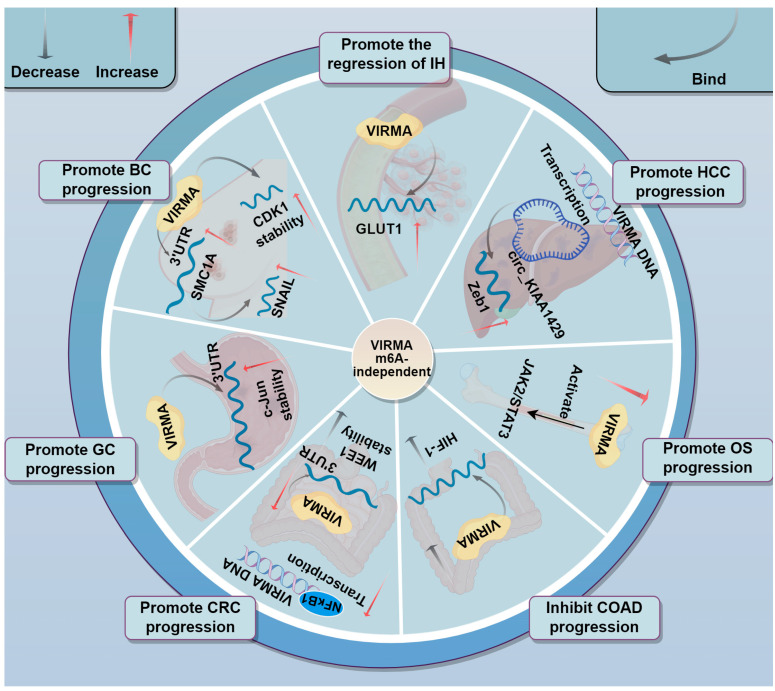
Involvement of VIRMA in tumor progression through m6A-independent mechanisms.

The function of VIRMA is not limited to regulating gene expression through m6A-dependent mechanisms (Figure 5). Its action as an RNA binding protein independent of m6A modification is also found in many cancers.

Infantile hemangioma (IH) is a common vascular tumor with a specific natural course, often involving the skin, subcutaneous tissues, and even internal organs. GLUT1, a marker of facultative stem cells, is closely related to the specific course of IH [117]. In IH, VIRMA could upregulate GLUT1, promoting the conversion of hemangioma endothelial cells into stem cells. Through RIP experiments, researchers found that VIRMA acts on GLUT1 in an m6A-independent manner. Moreover, the weakening of the endothelial cell phenotype and the promotion of conversion to stem cells align with the spontaneous regression process of IH to fibrofatty tissue, suggesting that VIRMA may promote the regression of IH [118]. CDK1 plays a key role in regulating the cell cycle and is closely related to cancer progression [119]. In BC, VIRMA directly interacted with CDK1 transcripts and prolonged their half-life, but VIRMA knockdown did not change m6A-modified CDK1 mRNA level, indicating VIRMA regulates CDK1 mRNA stability in an m6A-independent manner [83]. In another study, the VIRMA/SMC1A/SNAIL axis was found to regulate BC metastasis. VIRMA directly bound to the SMC1A mRNA 3′UTR in an m6A-independent manner, stabilizing its expression. SMC1A then positively regulated SNAIL expression, which triggered EMT in BC cells by inhibiting cell adhesion proteins, promoting BC metastasis. Notably, the short (N-terminal) isoform of VIRMA was found overexpressed in this study, rather than the full-length isoform [21,120]. Similar to the interaction with SMC1A, VIRMA was demonstrated to directly bind the c-Jun mRNA 3′UTR in an m6A-independent manner, stabilizing its mRNA and affecting the growth of GC cells [86]. Furthermore, VIRMA also promoted cancer progression in an m6A-independent manner in CRC. A study by Ma et al. found that VIRMA could bind the 3′UTR of the tumor suppressor gene *WEE1* in an m6A-independent manner, decreasing the stability of WEE1 mRNA, thereby downregulating WEE1 expression and promoting CRC progression. NFκB1, a transcription factor in the NFκB1 family, was found to bind the VIRMA promoter. Interestingly, butyrate, a metabolite of the gut microbiota, can inhibit NFkB1 expression, further decreasing VIRMA levels [84,121].

However, in some studies, whether VIRMA functions dependent or independent of m6A is not clear. Hypoxia-inducible factor 1 (HIF-1), an oxygen-regulated transcription factor, plays a critical role in cancer progression. In colon adenocarcinoma (COAD), silencing VIRMA blocked the HIF-1 signaling pathway, inhibiting the proliferation, migration, invasion, and proptosis of colon cancer cells. However, whether VIRMA plays a role in this process through an m6A-independent mechanism remains to be further studied [37]. In OS, upregulation of VIRMA was significantly associated with poor prognosis in patients. The upregulation of VIRMA activated the JAK2/STAT3 signaling pathway, promoting the malignant phenotype progression of OS cells. However, whether this process involved m6A modification remains unclear [40]. Moreover, Wang et al. discovered that the circRNA hsa_circ_0084922 (named circ_KIAA1429), transcribed from VIRMA, was upregulated in liver cancer tissues and cell lines, promoting liver cancer cell migration, invasion, and EMT. The study showed that Zeb1 was a downstream target of circ_KIAA1429, and circ_KIAA1429 promoted liver cancer progression by targeting Zeb1 [85].

These studies indicate VIRMA’s dual role as both an m6A methylation regulator and a direct RNA-interacting factor in tumors, which offers key evidence for the multidimensional regulatory network in cancer development.

## 4. Upstream Regulatory Mechanisms of VIRMA

**Figure 6:**
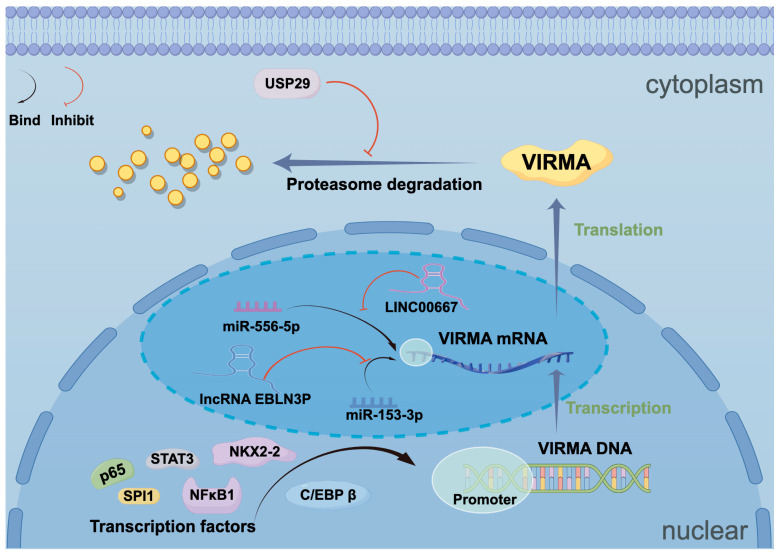
Upstream regulatory mechanisms of VIRMA. Multiple transcription factors (P65, STAT3, NKX2-2, SPI1, and NFκB1, C/EBPβ) enhance VIRMA promoter activity and transcription. lncRNA EBLN3P promotes VIRMA expression by competitively binding miR-153-3p, while VIRMA reciprocally enhances EBLN3P expression in an m6A-dependent manner. A similar regulatory mechanism exists between LINC00667, miR-556-5p and VIRMA. At the protein level, USP29 stabilizes VIRMA through deubiquitylation.

Transcription factors, non-coding RNAs, and ubiquitin-related enzymes can regulate the expression of VIRMA at multiple levels (Figure 6).

### 4.1. Transcription Factors

The study demonstrated that the transcription factor NKX2-2 is positively correlated with VIRMA and may serve as a specific upstream regulator of VIRMA in ES. Additionally, the study revealed that STAT3 can bind conservatively to the promoter of VIRMA [77]. Furthermore, the transcription factor NFκB1 could bind to the VIRMA promoter, upregulating its expression in CRC [84]. In OC, the transcription factor SPI1 regulated VIRMA transcription by binding to its DNA motif (AAGGAAGT) [45]. In PDAC, the transcription factor C/EBP β enhanced VIRMA promoter activity, thereby upregulating its expression [73]. The VIRMA promoter contains a p65 binding motif, and in gastric cancer, p65 upregulates VIRMA expression by binding to its promoter [71].

### 4.2. Non-Coding RNAs

LncRNA-miRNA networks also play a role in the regulation of VIRMA. In CRC, lncRNA EBLN3P competitively bound to miR-153-3p through a ceRNA network, promoting VIRMA expression, while VIRMA, in an m6A-dependent manner, enhanced the expression of lncRNA EBLN3P, forming a positive feedback loop. Similarly, in BC, a positive feedback regulatory mechanism also exists between lncRNA LINC00667, miR-556-5p, and VIRMA [75,81].

### 4.3. Ubiquitin-Related Enzymes

A study by Li et al. demonstrated that USP29, a member of the ubiquitin-specific protease (USP) family, stabilized VIRMA protein through deubiquitylation in CRC. VIRMA, in turn, enhanced SOX8 mRNA stability via m6A modification, promoting tumor progression [38].

## 5. The Potential of VIRMA in Cancer Diagnosis and Therapy

VIRMA serves as a core component of the m6A methyltransferase complex, demonstrating characteristic overexpression and tumor-specific subcellular localization across multiple malignancies. Its expression levels show strong correlations with tumor aggressiveness and poor clinical outcomes, positioning it as a promising diagnostic biomarker and therapeutic target (Table 2). Studies reveal VIRMA’s pan-cancer significance, with aberrant overexpression observed in over 20 cancer types that associate with chromosome 8q21.3-24.3 amplification [31]. Pathological analyses consistently demonstrate positive correlations between VIRMA expression and advanced TNM staging, lymph node metastasis, and distant metastasis, while showing negative associations with overall survival [24,39]. The protein exhibits distinct subcellular distribution patterns—predominantly nuclear in solid tumors such as hepatocellular and pancreatic carcinomas, yet cytoplasmically enriched in specific cancers including breast cancer, reflecting its functional heterogeneity across different tumor microenvironments and offering potential for molecular subtyping [19,23].

Extensive research has elucidated VIRMA’s dual regulatory mechanisms in tumor progression through both m6A-dependent and independent pathways. In the M6A-dependent pathway, VIRMA regulates key oncogenes (e.g., *FOXM1*, *HK2*) and tumor suppressor genes (e.g., *RND3*, *BTG2*), affecting the proliferation, metastasis, metabolic reprogramming, and drug resistance of tumor cells [18,24,39]. Additionally, VIRMA facilitates immune evasion within the tumor microenvironment via the KLF1-PD-L1 signaling axis [58]. The m6A-independent mechanism involves direct binding to 3′UTRs of specific mRNAs (CDK1, c-Jun) to regulate their stability [83,86]. These findings collectively demonstrate VIRMA’s multifaceted therapeutic potential across various intervention levels.

Current therapeutic strategies targeting VIRMA mainly focus on three aspects. One involves small-molecule inhibitor development, such as rucaparib, which binds to key residues LYS1029 and ASN1088 of VIRMA to inhibit its function [42]. Another strategy is gene therapy. CRISPR-Cas9-mediated knockout of VIRMA has shown anti-tumor effects in Ewing sarcoma models [77]. Future approaches may include targeted knockout or silencing of VIRMA using nanoparticle delivery systems, offering new possibilities for cancer treatment. In addition, combination therapies of VIRMA inhibition with ferroptosis inducers or immune checkpoint inhibitors have shown synergistic effects [57,58]. These preliminary findings lay a foundation for future exploration of VIRMA in cancer therapy.

Future studies should emphasize interdisciplinary collaboration. Integrating cutting-edge technologies such as single-cell sequencing and spatial transcriptomics may help to map the detailed regulatory role of VIRMA in tumor heterogeneity. AI-assisted molecular docking and virtual screening could accelerate the development of highly selective inhibitors. Organoid and patient-derived xenograft (PDX) models may better simulate clinical drug responses. It is also important to explore the potential of VIRMA in early cancer detection and treatment monitoring, including its value as a biomarker in exosomes or circulating tumor cells for liquid biopsy applications.

**Table 2:** Clinical significance of VIRMA and its relationship with patient survival.

Cancer Type	Clinical Significance	Targets	Survival Association	Ref.
HNSCC	Oncogene	UBR5	Not explored	[35]
NPC	Oncogene	PTGS2; LINC00839; E2F7	Worst OS	[32,46,47]
OSCC	Oncogene	inhibitor A; PGK1; CA9	Worst OS	[33,48,49]
NSCLC	Oncogene	PXFP1; WTAP; BTG2; DAPK3; MUC3A; ARHGAP30; LINC01106; P53; KLF1; HOXA1; MAP3K2	Worst OS	[34,50,51,52,53,54,55,56,57,58,59]
HCC	Oncogene	RND3; HPN; circDLC1; GATA3 pre-mRNA; ID2; HK1; SLC7A11; CCR9; HSPG2; HBx; Zeb1	Worst OS	[18,23,60,61,62,63,64,65,66,67,85,122]
ICC	Oncogene	CCL3; TMED2P; ARD3B; SIRT1	Worst OS	[68,69]
GC	Oncogene	RASD1; LINC00958; FOXM1; P65; c-Jun	Worst OS	[36,70,71,72,86]
PDAC	Oncogene	STRA6; SLC43A2; C/EBP β	WorsetOS	[24,73]
CRC	Oncogene	lncRNA POU6F3-AS1; USP29; SOX8; SIRT1; miR-53-3p; lncRNA EBLN3P; HIF-1; NFκB1; WEE1	Worst OS	[20,37,38,74,75,84]
OS	Oncogene	CDK1; CCNA2; CCNB1; JAK-STAT	Worst OS	[40,76]
ES	Oncogene	NKX2-2; STAT3	Not explored	[77]
MM	Oncogene	FOXM1	Worst OS	[39]
CML	Oncogene	RAB27B	Not explored	[42]
DLBCL	Oncogene	CHST11	Worst OS	[41]
BC	Oncogene	HAS2; KIF15; TFAP2A; DDR1; NEAT1; miR-556-5p; LINC00667; SMC1A; CDK1	Worse OS	[19,21,78,79,80,81,83]
TGCTs	Oncogene	Not explored	Not explored	[43]
CC	Oncogene	BTG2; LARP1	Worst OS	[44,82]
OC	Oncogene	SPI1; ENO1	Worst OS	[45]

## 6. Conclusions

In summary, VIRMA is a key molecule with both fundamental research value and strong potential for clinical translation. Advances in understanding its molecular mechanisms, along with continuous technological innovation, are expected to drive the development of VIRMA-based diagnostic tools and targeted therapies toward clinical application. These strategies may offer new options for cancer diagnosis and treatment. Progress in this field will require close collaboration among basic researchers, clinicians, and the pharmaceutical industry to bridge the gap between laboratory discoveries and clinical practice.

## Figures and Tables

**Table 1:** Expression, regulatory mechanisms, and biological functions of VIRMA in various cancers.

Cancer Type	Expression of VIRMA	m6A-Dependent Regulation	Upstream Regulation	Downstream Targets	Function	In Vivo Study	Ref.
HNSCC	Up	Yes.	/	UBR5	Promote cancer progression; influence mRNA stability	/	[35]
NPC	Up	Yes.	/	PTGS2	Promote cancer progression; influence mRNA stability	Yes	[46]
	Up	Yes	/	LINC00839	Promote cancer progression	Yes	[47]
	Up	Yes	/	E2F7	Promote cancer progression	Yes	[32]
OSCC	Up	Yes	/	inhibitor A	Promote cancer progression	Yes	[48]
	Up	Yes	/	PGK1	Promote cancer progression; mediate resistance to ferroptosis; affect cancer metabolism	Yes	[33]
	Up	Yes	/	CA9	Promote cancer progression	Yes	[49]
NSCLC	Up	Yes	/	PXFP1	Promote cancer progression	/	[50]
	Up	Yes	/	WTAP	Promote cancer progression, affecting drug resistance.	Yes	[51]
	Up	Yes	/	BTG2	Promote cancer progression; influence mRNA stability	Yes	[52]
	Up	Yes	/	DAPK3	Promote cancer progression	Yes	[53]
	Up	Yes	/	MUC3A	Promote cancer progression by influencing the cell cycle.	/	[54]
	Up	Yes	/	ARHGAP30	Promote cancer progression	/	[55]
	Up	Yes	/	LINC01106	Promote cancer progression	Yes	[56]
	Up	Yes	/	P53	Promote cancer progression; mediating resistance to ferroptosis	Yes	[57]
	Up	Yes	/	KLF1	promoting cancer cell proliferation and metastasis; mediating immune escape	/	[58]
	Up	Yes	/	HOXA1	Promote cancer progression, affecting drug resistance.	/	[59]
	Up	Yes	/	MAP3K2	Promote cancer progression, affecting drug resistance.	Yes	[34]
HCC	Up	Yes	/	RND3	Promote cancer progression; influence mRNA stability	Yes	[18]
	Up	Yes	/	HPN	Promote cancer progression	/	[60]
	Up	Yes	/	circDLC1	Promote cancer progression	Yes	[61]
	Up	Yes	/	GATA3 pre-mRNA	Promote cancer progression; influence mRNA stability	Yes	[23]
	Up	Yes	/	ID2	Promote cancer progression	/	[62]
	Up	Yes	/	HK1	Promote cancer progression, affecting drug resistance and affecting cancer metabolism.	Yes	[63]
	Up	Yes	/	SLC7A11	Promote cancer progression; mediating resistance to ferroptosis	Yes	[64]
	Up	Yes	/	CCR9	Promote cancer progression, affecting drug resistance.	Yes	[65]
	Up	Yes	HBx	HSPG2	Promote cancer progression; influence mRNA stability	/	[66]
	Up	Yes	/	/	Promote cancer progression, affecting drug resistance.	Yes	[67]
ICC	Up	Yes	/	TMED2P; ARD3B	Promote cancer progression	Yes	[68]
	Up	Yes	CCL3	SIRT1	Promote cancer progression; influence mRNA stability and influencing cell cycle.	Yes	[69]
GC	Up	Yes	/	RASD1	Promote cancer progression; influence mRNA stability; influence cell cycle	Yes	[36]
	Up	Yes	/	LINC00958	Promote cancer progression, affecting cancer metabolism.	Yes	[70]
	Up	Yes	P65	FOXM1	Promote cancer progression, affecting drug resistance.	Yes	[71]
	Up	Yes	/	FOXM1	Promote cancer progression, affecting drug resistance.	/	[72]
PDAC	Up	Yes	/	STRA6	Promote cancer progression, affecting cancer metabolism.	Yes	[24]
	Up	Yes	C/EBP β	SLC43A2	Promote cancer progression; influence mRNA stability	Yes	[73]
CRC	Up	Yes	/	lncRNA POU6F3-AS1	Promote cancer progression	/	[74]
	Up	Yes	USP29	SOX8	Promote cancer progression	Yes	[38]
	Up	Yes	/	SIRT1	Promote cancer progression; influence mRNA stability	Yes	[20]
	Up	Yes	miR-153-3p	lncRNA EBLN3P	Promote cancer progression; mediating resistance to ferroptosis	/	[75]
OS	Up	Yes	/	CDK1; CCNA2; CCNB1	Promote cancer progression	Yes	[76]
ES	Up	Yes	NKX2-2 STAT3	STAT3	Promote cancer progression	Yes	[77]
MM	Up	Yes	/	FOXM1	Promote cancer progression, affecting cancer metabolism.	Yes	[39]
CML	Up	Yes	/	RAB27B	Promote cancer progression, affecting drug resistance.	Yes	[42]
DLBCL	Up	Yes	/	CHST11	Promote cancer progression by influencing the cell cycle.	Yes	[41]
BC	Up	Yes	/	HAS2	Promote cancer progression; influence mRNA stability	Yes	[19]
	Up	Yes	/	KIF15	Promote cancer progression; influence mRNA stability	Yes	[78]
	Up	Yes	/	TFAP2A; DDR1	Promote cancer progression, mediating immune escape	Yes	[79]
	Up	Yes	/	NEAT1	Promote cancer progression	Yes	[80]
	Up	Yes	miR-556-5p	LINC00667	Promote cancer progression	/	[81]
TGCTs	Up	Yes	/	/	Promote cancer progression	Yes	[43]
CC	Up	Yes	/	BTG2	Promote cancer progression; influence mRNA stability	/	[44]
	Up	Yes	/	LARP1	Promote cancer progression; influence mRNA stability.	Yes	[82]
OC	Up	Yes	SPI1	ENO1	Promote cancer progression, affecting cancer metabolism.	Yes	[45]
OS	Up	Not explored	/	JAK-STAT	Promote cancer progression	Yes	[40]
CRC	Up	Not explored	/	HIF-1	Promote cancer progression	Yes	[83]
	Up	No	NFκB1	WEE1	Promote cancer progression	Yes	[84]
BC	Up	No	/	SMC1A	Promote cancer progression	Yes	[21]
	Up	No	/	CDK1	Promote cancer progression, by influencing the cell cycle.	Yes	[83]
HCC	Up	No	/	Zeb1	Promote cancer progression	/	[85]
GC	Up	No	/	c-Jun	Promote cancer progression, by influencing the cell cycle.	Yes	[86]

## Data Availability

Not applicable.

## Abbreviations

m6A	N6-methyladenosine
VIRMA	Virus-like m6A methyltransferase associated
5′-UTR	5′ untranslated region
3′-UTR	3′ untranslated region
METTL3	Methyltransferase-like 3
METTL14	Methyltransferase-like 14
WTAP	Wilms’ tumor 1-associating protein
RBM15	RNA-binding motif protein 15
BC	Breast cancer
PDAC	Pancreatic ductal adenocarcinoma
MAC	m6A-METTL complex
MACOM	m6A-METTL- associated complex
dsDNA	double-stranded DNA
M3-M14-W-V	METTL3-METTL14-WTAP-VIRMA quaternary complex
RBPs	RNA-binding proteins
IF2	Translation initiation factor 2
HNSCC	Head and neck squamous cell carcinoma
NPC	Nasopharyngeal carcinoma
OSCC	Oral squamous cell carcinoma
NSCLC	Non-small cell lung cancer
HCC	Hepatocellular carcinoma
ICC	Intrahepatic cholangiocarcinoma
GC	Gastric cancer
CRC	Colorectal cancer
MM	Multiple myeloma
OS	Osteosarcoma
DLBCL	Diffuse large B-cell lymphoma
CML	Chronic myeloid leukemia
TGCTs	Testicular germ cell tumors
CC	Cervical cancer
OC	Ovarian cancer
ES	Ewing’s sarcoma
CCL3	Macrophage Inflammatory Protein-1 alpha, MIP-1α
HBV	Hepatitis B virus
HBx	Hepatitis B virus X protein
INHBA	Inhibin A
HAS2	Hyaluronan synthase 2
HPN	Hepsin
DAPK3	Death-Associated Protein Kinase 3
RXFP1	Relaxin Family Peptide Receptor 1
LUAD	Lung adenocarcinoma
LncRNAs	Long non-coding RNAs
VIRMA FL	Full-length isoform of VIRMA
circRNA	Circular RNA
EMT	Epithelial-mesenchymal transition
GLUT1	Glucose transporter-1
HK	Hexokinase
ENO1	Alpha-enolase
ECAR	Extracellular acidification rate
OCR	Oxygen consumption rate
LDHA	Lactate dehydrogenase A
GSH	Glutathione
ROS	Reactive oxygen species
ECM	Extracellular matrix
RTK	Receptor tyrosine kinase
SHBs	Hepatitis B virus surface small antigen
ER	Endoplasmic reticulum
UPR	Unfolded protein response
IH	Infantile Hemangioma
HIF-1	Hypoxia-inducible factor 1
COAD	Colon adenocarcinoma
USPs	Ubiquitin-specific protease
GDSC	Cancer drug sensitivity genomics
